# Bone Bricks: The Effect of Architecture and Material Composition on the Mechanical and Biological Performance of Bone Scaffolds

**DOI:** 10.1021/acsomega.1c05437

**Published:** 2022-02-22

**Authors:** Evangelos Daskalakis, Boyang Huang, Cian Vyas, Anil A. Acar, Fengyuan Liu, Ali Fallah, Glen Cooper, Andrew Weightman, Gordon Blunn, Bahattin Koç, Paulo Bartolo

**Affiliations:** † School of Mechanical, Aerospace and Civil Engineering, University of Manchester, ManchesterM13 9PL, U.K.; ‡ Integrated Manufacturing Technologies Research and Application Center, Sabanci University, Tuzla 34956, Istanbul, Turkey; § SUNUM Nanotechnology Research Center, Sabanci University, Tuzla 34956, Istanbul, Turkey; ∥ Faculty of Engineering and Natural Sciences, Sabanci University, Tuzla 34956, Istanbul, Turkey; ⊥ School of Pharmacy and Biomedical Sciences, University of Portsmouth, PortsmouthPO1 2DT, U.K.; # Department of Mechanical Engineering, School of Civil, Aerospace and Mechanical Engineering, Faculty of Engineering, University of Bristol, Bristol BS8 1TR, U.K.; ∇ Singapore Centre for 3D Printing, School of Mechanical and Aerospace Engineering, Nanyang Technological University, 639798, Singapore

## Abstract

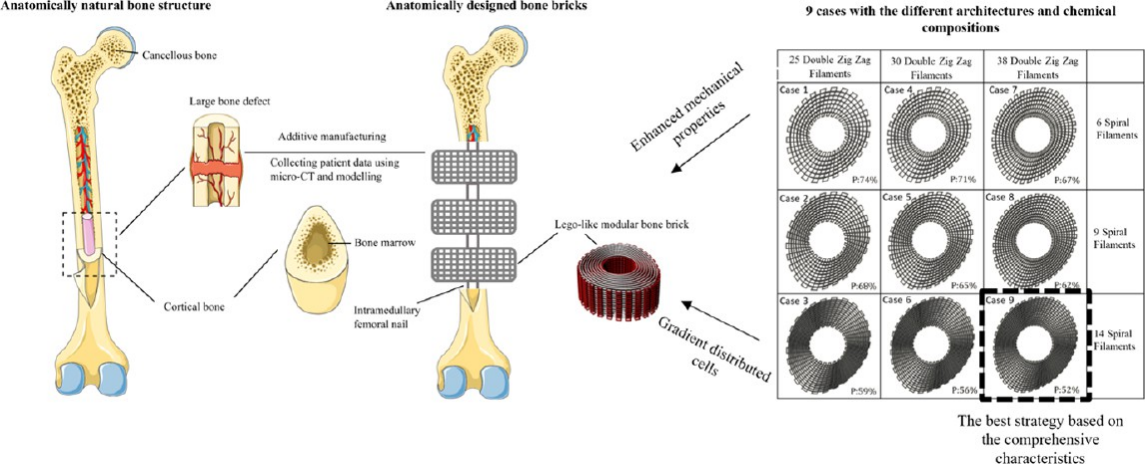

Large bone loss injuries require high-performance scaffolds with an architecture and material composition resembling native bone. However, most bone scaffold studies focus on three-dimensional (3D) structures with simple rectangular or circular geometries and uniform pores, not able to recapitulate the geometric characteristics of the native tissue. This paper addresses this limitation by proposing novel anatomically designed scaffolds (bone bricks) with nonuniform pore dimensions (pore size gradients) designed based on new lay-dawn pattern strategies. The gradient design allows one to tailor the properties of the bricks and together with the incorporation of ceramic materials allows one to obtain structures with high mechanical properties (higher than reported in the literature for the same material composition) and improved biological characteristics.

## 1 Introduction

Bone tissue can heal itself in the case of small defects. However, in the case of large-scale fractures, such as bone loss, nonunion, and tumor removal, bone regeneration is limited.^1−3^ Moreover, due to the size of the fractured area and open wounds, bone is vulnerable to infections due to the presence of pathogens that can lead to the delay of the healing process, inflammation, and creation of chronic osteomyelitis during the injury time, the surgery, or the recovery process.^4,5^

Several surgical techniques have been developed to address these large bone fractures but they require complex and multiple procedures, leading to a high rate of complications and morbidity. In a large number of cases, amputation is the only alternative (90% of the cases), but with a high loss of the damaged limb.^6−8^ Bone-shortening and distraction osteogenesis (internal fixation) are other alternative techniques.^9−11^

Bone-shortening technique is used for bone defects lower than 8 cm of size, but it needs a long hospitalization period and multiple surgery procedures and causes chronic pain and risks such as microbial infection, osteomyelitis, blood vessel and nerve damage, and poor bone healing.^12,13^ The use of prosthesis allows surgeons to replace the fractured limb using megaprosthesis, but this approach is performed only in special cases such as malignant tumors, nonneoplastic conditions, major trauma, or end-stage revision arthroplasty and extensive metastatic diseases.^14,15^ Internal fixation techniques, such as plates or intramedullary nail, are used to stabilize bone gaps. However, these techniques increase the risk of complications due to infections, leading to larger defects.^16^ Distraction osteogenesis involves a modular-ring external fixator (Ilizarov apparatus) that stimulates the blood flow, allows early bearing, and creates good-quality bone tissue.^17,18^ However, it is a technique that requires complex procedures leading to infections, nerve and blood vessel damage, chronic pain, and long hospitalization.^19,20^ Masquelet and Begue^21^ developed a new concept based on a two-stage technique treating bone injuries in infected and noninfected conditions. First, a combination of cement spacer and induced membrane is used to fill the defected area.^22^ Second, a biological implant replaces the cement spacer, which is impermeable, biological, and vascular-active.^22^ However, autografts present some limitations such as morbidity in the donor site, pain, long hospitalization time, limited quantity, needing general anesthesia during the surgery, extended nonweight-bearing, graft hypertrophy, and the risk of hematoma and deep infection.^23,24^ Therefore, it is clear that novel bone replacements are highly demanded and must be bioactive, biodegradable, biocompatible, bespoke, resistant to infections, cost-effective, and have high porosity to enable cell attachment and vascularization, providing similar mechanical and biological properties as bone tissue.^25,26^

The use of synthetic grafts (scaffolds) produced using biocompatible and biodegradable materials emerged as a potential alternative to current clinical approaches. Moreover, these grafts can be easily produced using additive manufacturing, a group of techniques that produce objects by adding materials layer-by-layer, capable of providing control on the size, shape, and porosity of the grafts.^27,28^ However, scaffolds produced by additive manufacturing usually exhibit regular shapes (square and circle scaffolds), thus not recapitulating the anatomical features of native bone and its pore size gradient. It is known that large pore size promotes vascularization, allowing better exchange of nutrients and oxygen and tissue infiltration inside the scaffold, while smaller pore sizes enhance the mechanical properties of the scaffold.^29^ However, it is difficult to balance these properties only using scaffolds with regular shapes, such as the scaffolds reported in the literature. Nevertheless, to our best knowledge, there are no publications reporting the design of scaffolds with a gradient porosity and pore size and, more importantly, being anatomically designed, thus sharing a similar structure as natural bone.^30^

**Figure 1:**
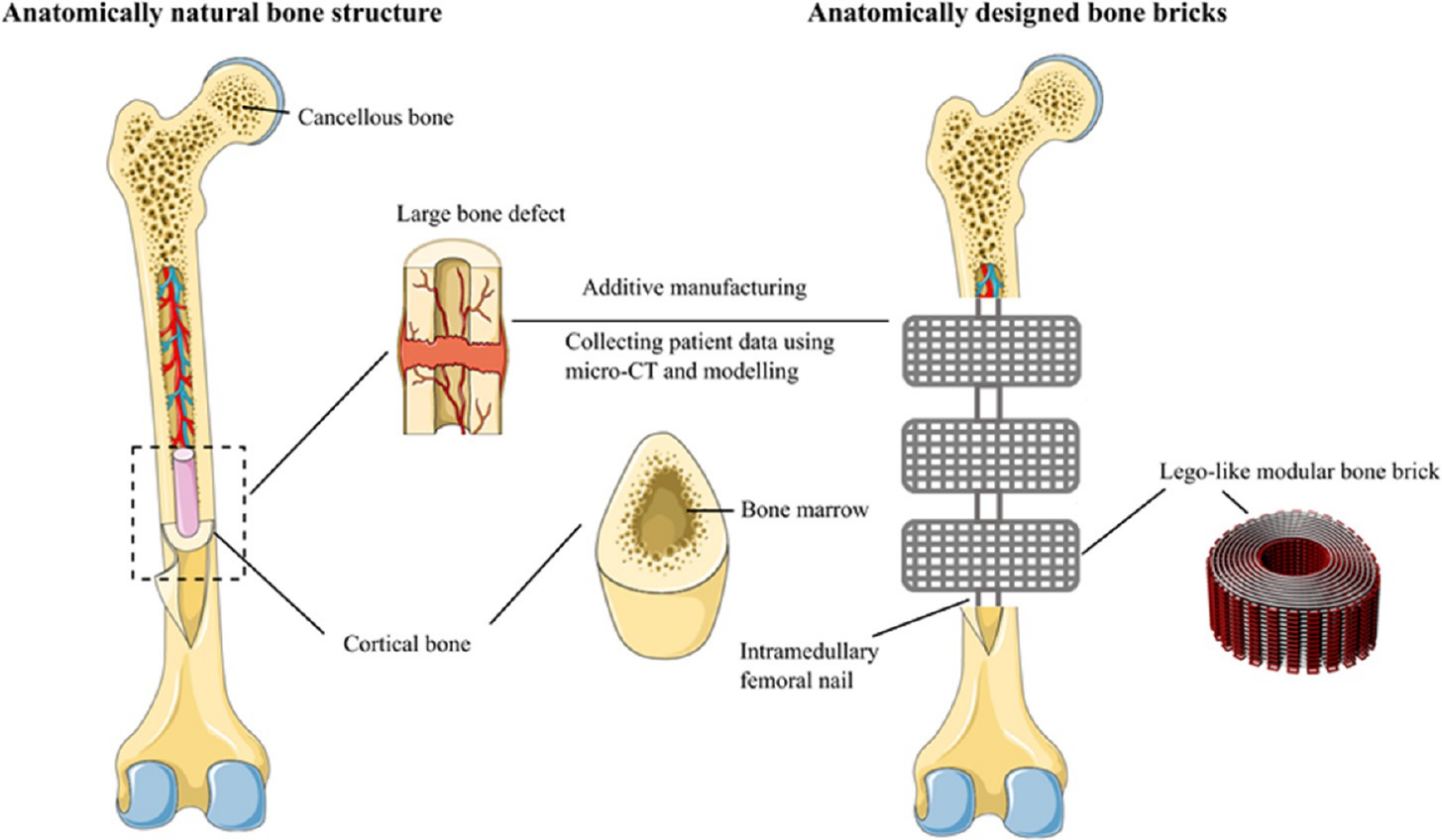
Bone anatomy and the bone bricks concept investigated in this work.

Bone tissue is a highly hierarchical structure, composed of water, mineralized collagen fibril bundles (ossein), and noncollagen proteins.^31^ Bone tissue comprises two main regions, being characterized by a nonhomogeneous structure consisting of a dense cortical region and a highly porous cancellous tissue.^32−34^ The first region consists of Haversian canals and osteons, while the second region consists of a spongy bone with the porosity ranging from 75 to 85%.^31−34^ Cortical bone presents a more compact structure with the porosity ranging between 5 and 10%.^32−35^ As a consequence of this structure, bone presents a gradient of pore sizes, ranging from 100 to 600 μm, which differ based on the age and sex, with the more compact and dense regions being responsible for supporting load bearings and the less dense and more porous regions playing an important role in the exchange of minerals.^34,35^ The development of geometrically graded porous scaffolds recapitulating bone anatomical shape is the main aim of this manuscript (Figure 1).

Through a project entitled “bone bricks: low cost-effective modular osseointegration prosthetics for large bone loss surgical procedures”, funded by EPSRC/GCRF (Engineering and Physical Sciences Research Council/Global Challenges Research Fund), the authors are investigating novel low-cost modular prosthetic solutions for the treatment of large-scale bone tissue defects, enabling the salvation of lower limbs. The immediate application was to treat the Syrian refugees in Turkey, suffering from large bone fractures due to traumatic injuries and explosions. Through this project, the authors are proposing a strategy based on the combined use of an external fixation and an internal prosthetic implant (bone bricks) that will connect in a “lego-like” way, filling the damaged area. The aim is to improve patient outcome, reduce hospitalization time, and avoid painful limb lengthening.

In a previous study, we successfully generated computer-aided design (CAD) models based on the micro-CT scan of patient bones, based on anthropometric data collected from a total of 1198 males and 1059 females, capturing gender differences.^30^ In this paper, we investigate different configurations of anatomically designed bone bricks presenting different porosity gradients. Bone bricks were produced using different printing strategies and combinations of polycaprolactone (PCL), hydroxyapatite (HA), and tricalcium phosphate (TCP). The gradient design allows one to tailor the properties of the bricks and together with the incorporation of ceramic materials allows one to obtain three-dimensional (3D) porous structures with high mechanical properties (higher than reported in the literature for the same material composition) and improved biological characteristics.

## 2 Materials and Methods

### 2.1 Materials

Poly-ε-caprolactone (PCL) (CAPA 6500, Mw = 50 000 Da) was provided by Perstorp Caprolactones (Cheshire, U.K.) in a pellet shape. Hydroxyapatite (HA) (Mw = 502.31 r/mol, MP = 1100 °C) was provided by Sigma-Aldrich (St. Loius) in the nanopowder form (<200 nm particle size), and β-tricalcium phosphate (TCP) (Mw = 310.18 r/mol, MP = 1391 °C) was provided by Sigma-Aldrich (St. Loius) in the powder form (ranging from 20 to 30 μm). PCL-based composite blends containing different bioceramic concentrations (10 wt % HA, 15 wt % HA, 20 wt % HA, 10 wt % TCP, 15 wt % TCP, 20 wt % TCP, and 10 wt % HA plus 10 wt % TCP) were prepared through melt blending. Briefly, PCL was weighed with a high-precision (precision of 0.0001) electronic scale. The pellets were then melted inside a porcelain plate at a temperature of 150 °C prior to ceramic particle addition. The uniform distribution of ceramic particles in the composite mixtures was ensured by mixing them for at least an hour.

### 2.2 Bone Brick Production

**Figure 2:**
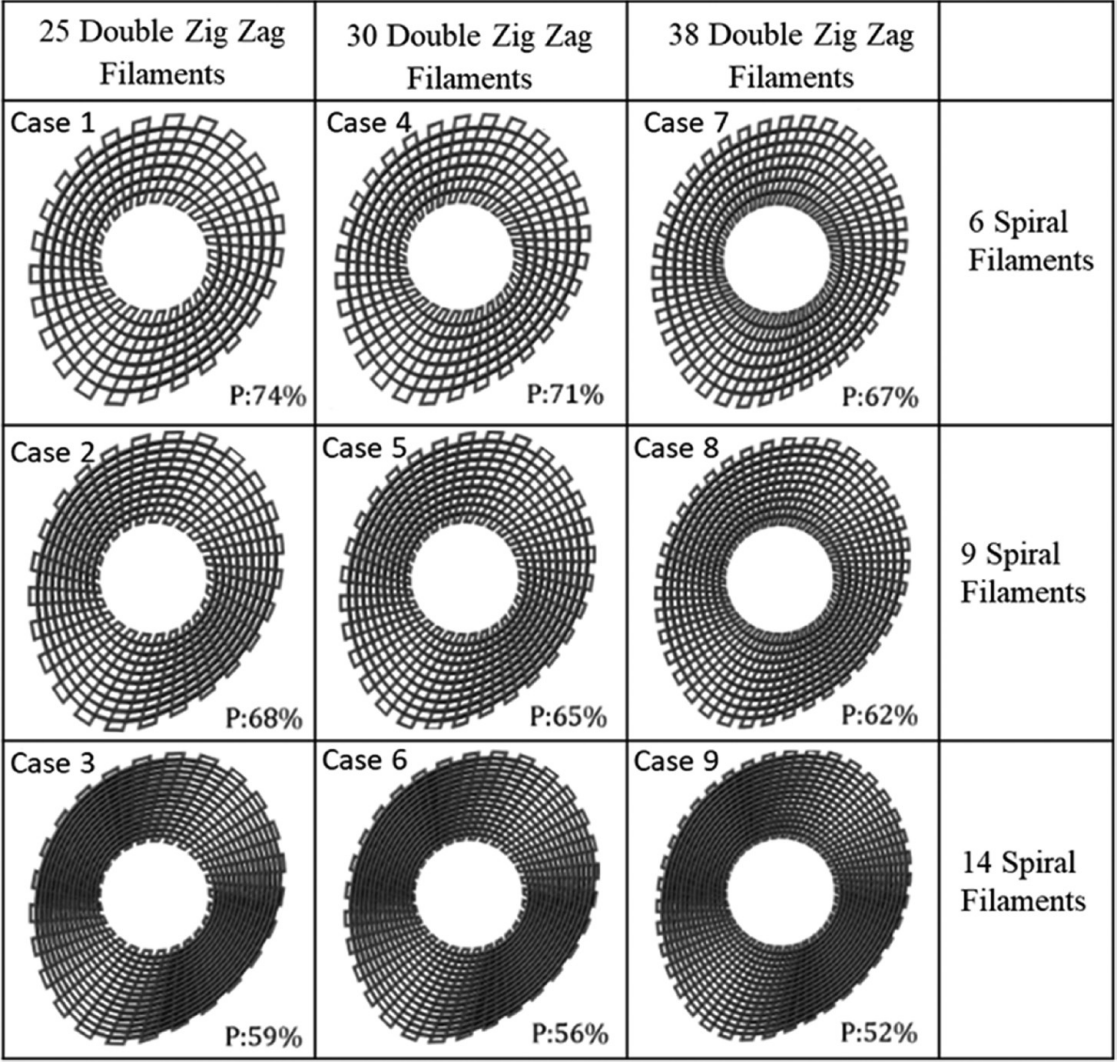
Bone brick designs based on anthropometric and computer-aided design strategies (P stands for porosity).

A screw-assisted extrusion-based additive manufacturing 3D Discovery system (RegenHU, Switzerland) machine was used for bone brick fabrication. A continuous path algorithm was created,^30^ with the combination of anthropometric measurements and computer-aided design (CAD), using zig-zag double filaments (25, 30, and 38) and spiral filaments (6, 9, and 14) to create nine different designs of bone bricks with varying porosities (52–74%) with overall dimensions of 31 mm × 26.7 mm × 10 mm (length × width × height) (Figure 2). Bone bricks were fabricated considering the following processing parameters: 90 °C of melting temperature, 20 mm/s of deposition velocity, and 12 rpm of screw rotational velocity. The filaments were extruded using a 0.33 mm-diameter needle.

### 2.3 Morphological Characterization

A scanning electron microscopy (SEM) machine, FEI ESEM Quanta 200 (FEI Company), was used to investigate the morphological characteristics of fabricated bone bricks. Bone ricks were coated (gold coating) using the EMITECH K550X sputter coater (Quorum Technologies, U.K.) before imaging. The SEM images were analyzed by ImageJ (NIH) (10 measurements), allowing one to determine the pore size (PS), the filament width (FW), and the layer gap (LG).

### 2.4 Thermal Gravimetric Analysis (TGA)

A Netzsch TGA-differential thermal analysis (TGA–DTA) system (Netzsch, Germany) was used to investigate thermal degradation and to calculate the ceramic concentration in the bone bricks. The tests were conducted in an air atmosphere (50 mL/min) with the temperature ranging from 25 to 1000 °C at the rate of 10 °C/min. Each test was conducted twice using platinum pans.

### 2.5 Apparent Water Contact Angle (WCA)

**Figure 3:**
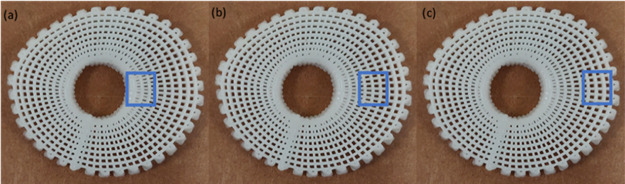
Different regions for the water contact angle tests: (a) inner region, (b) middle region, and (c) outer region.

Water contact angle (WCA) tests were carried out using the OCA 15 system (Data Physics, San Jose, CA). During the test, deionized water (4 μL of volume drop, 1 μL/s velocity), in the form of a droplet, was dropped on the surface of the bone bricks by a fixed pipet with the bone brick aligned with the camera and recorded with a high-speed framing camera. This way, it is possible to evaluate the effect of both material composition and bone brick architecture on the wettability. Two time points (0 and 20 s) were considered, and all tests were performed in triplicate considering three different regions on the bone bricks (Figure 3).

### 2.6 Fourier Transform Infrared (FTIR) Spectroscopy

Fourier transform infrared (FTIR) spectroscopy tests were conducted using the Nicolet iS 10 system (Thermo Scientific) to determine structural changes in both PCL and composite bone bricks. The transmittance was evaluated in the wavenumber ranging from 3500 to 500 cm^–1^ at room temperature.

### 2.7 Energy-Dispersive X-ray Spectroscopy (EDX)

The chemical composition of bone bricks before and after cell seeding was analyzed through energy-dispersive X-ray spectroscopy (EDX), determining the concentration of calcium (Ca), carbon (C), oxygen (O), and phosphorous (P). The analysis was performed using the SEM FEG FEI Quanta 200 (FEI Company). Bone bricks were gold-coated prior to imaging. The obtained SEM images were analyzed using Oxford AZtec software (Oxford Instruments, U.K.).

### 2.8 X-ray Diffraction (XRD)

X-ray diffraction (XRD) was used to investigate the crystalline patterns on PCL and composite bone bricks. Tests were performed using the D2 PHASER Bruker (Bruker D2 advance, Germany) with a copper anode source. The samples were flattened on a metal holder, and data was recorded with a 0.02–2θ° step for a total recording time of 75 min. The detector recording time was 1 s per point. A total of 4500 points were recorded.

### 2.9 Mechanical Characterization

Compression tests were performed using the INSTRON 3344 system (Instron, U.K.). Bone bricks were compressed with the use of a 2 kN load head and a 0.5 mm/min displacement rate, according to the ASTM D695-15 standards. The dimensions of the bone bricks were 31 mm × 26.7 mm × 10 mm (length × width × height), and tests were repeated 4 times. Force versus displacement curves obtained using Bluehill Universal Software (Instron, U.K.) were converted into stress–strain curves, and the compressive modulus was determined using GraphPad Prism software (GraphPad Software Inc., San Diego, CA).

### 2.10 In Vitro Study

Human adipose-derived stem cells (hADSCs) (STEMPRO, Invitrogen) (passage 5) were used to investigate cell attachment and spreading on the bone bricks. During cell culture, MesenPRO RSTM basal media, 2% (v/v) growth supplement, 1% (v/v) glutamine, and 1% (v/v) penicillin/streptomycin (Invitrogen) were used as nutrition supplement.

Bone bricks were placed into 50 mL tubes containing 80 wt % pure ethanol and 20 wt % distilled water for 4 h. Then, the liquid was poured out and the bone bricks were washed two times with the use of phosphate-buffered saline (PBS) to remove any dead microorganisms and dust particles. Bone bricks were left to dry for 24 h after the sterilization process. The dimensions of the bone bricks used for in vitro studies were 10 mm × 10 mm × 10 mm (length × width × height) to fit into the 24-well plates. The cell seeding process followed the manufacturer’s guidelines and a procedure previously reported.^35^ The recommended cell seeding density is 5000 cells/cm^2^, but to guarantee sufficient spatial growth and appropriate cell confluency, it was decided to use 50 000 cells/per well. Moreover, all samples were seeded with the same volume of cell suspensions (89 μL), allowing comparability between samples. After the cell seeding, the well plates were incubated for 2 h, followed by the addition of culture media to each well.

On days 1, 7, and 14, bone bricks were transferred to new well plates and assessed by the Alamar Blue (Sigma-Aldrich, U.K.) assay, which determines the cell metabolic activity and provides indirect information on cell attachment and proliferation.^36,37^

Briefly, Alamar Blue solution of 0.001 wt % (90 μl) was poured into each well and placed in an incubator for 4 h. A certain amount of sample solution (200 μL) was transferred into a 96-well plate and, with the use of a microplate reader Synergy HTX Multi-Mode Reader (BioTek), the fluorescence intensity of cells was calculated (530 nm excitation/590 nm emission wavelength). Then, bone bricks were washed with PBS and fresh media was added.

On day 14, the cell morphology was investigated through SEM imaging. For the fixation of cells on the bone bricks’ surface, a 10 wt % neutral buffered formalin (Sigma-Aldrich, Dorset, U.K.) was used for 30 min at room temperature. Then, bone bricks were rinsed twice in PBS. Following the fixation procedure, bone bricks were dehydrated into the well plates, with the use of graded ethanol mixed with deionized water (50, 60, 70, 80, 90, and two times of 100%). The two final steps were the use of 50/50 ethanol/hexamethyldisalazane (HDMS) (Sigma-Aldrich) (v/v) solution, followed by adding 100% HDMS and leaving the liquid to evaporate overnight under a hood. Each concentration of ethanol/deionized water and HDMS was used for 15 min.

Confocal laser scanning microscopy was used to study cell spreading and the anatomy of cells attached to the bone bricks. Alexa Fluor 488-conjugated phalloidin (Thermo Fisher Scientific, U.K.) and 4′,6-diamidino-2-phenylindole (DAPI) were used for staining, respectively, the actin cytoskeleton and nucleus of the cells. A Leica TCS SP5 (Leica, Milton Keynes, U.K.) confocal microscope was used for imaging of cells on the bone bricks.

### 2.11 Data Analysis

Mechanical and biological data are represented as mean ± standard deviation. Statistical analysis was conducted with the use of one-way analysis of variance (ANOVA) with Tukey’s post hoc test. Statistically significant differences were considered at *p < 0.05, **p < 0.01, ***p < 0.001, and ****p < 0.0001. TGA, XRD, and FTIR data were analyzed using Origin 2021 (OriginLab Corporation, Northampton, Massachusetts).

## 3 Results and Discussion

### 3.1 Morphological Analysis

**Figure 4:**
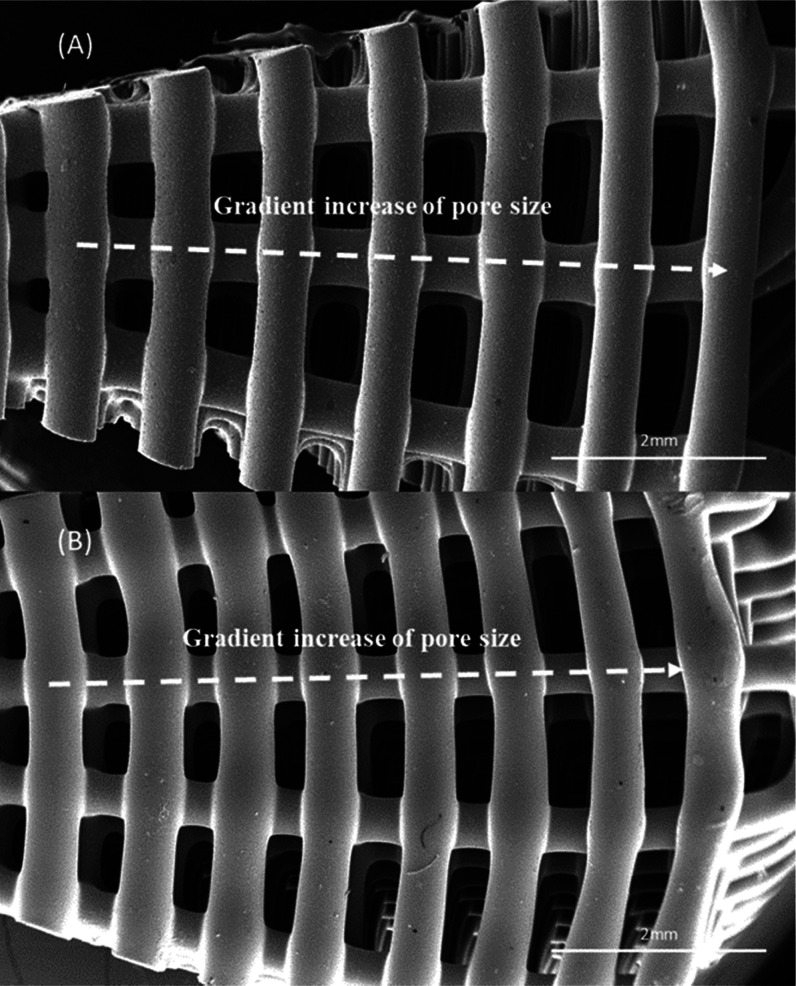
SEM images of (A) 15 wt % HA bone bricks (top view) corresponding to the architecture case 5 and (B) 15 wt % TCP bone bricks (top view) corresponding to the architecture case 6.

**Figure 5:**
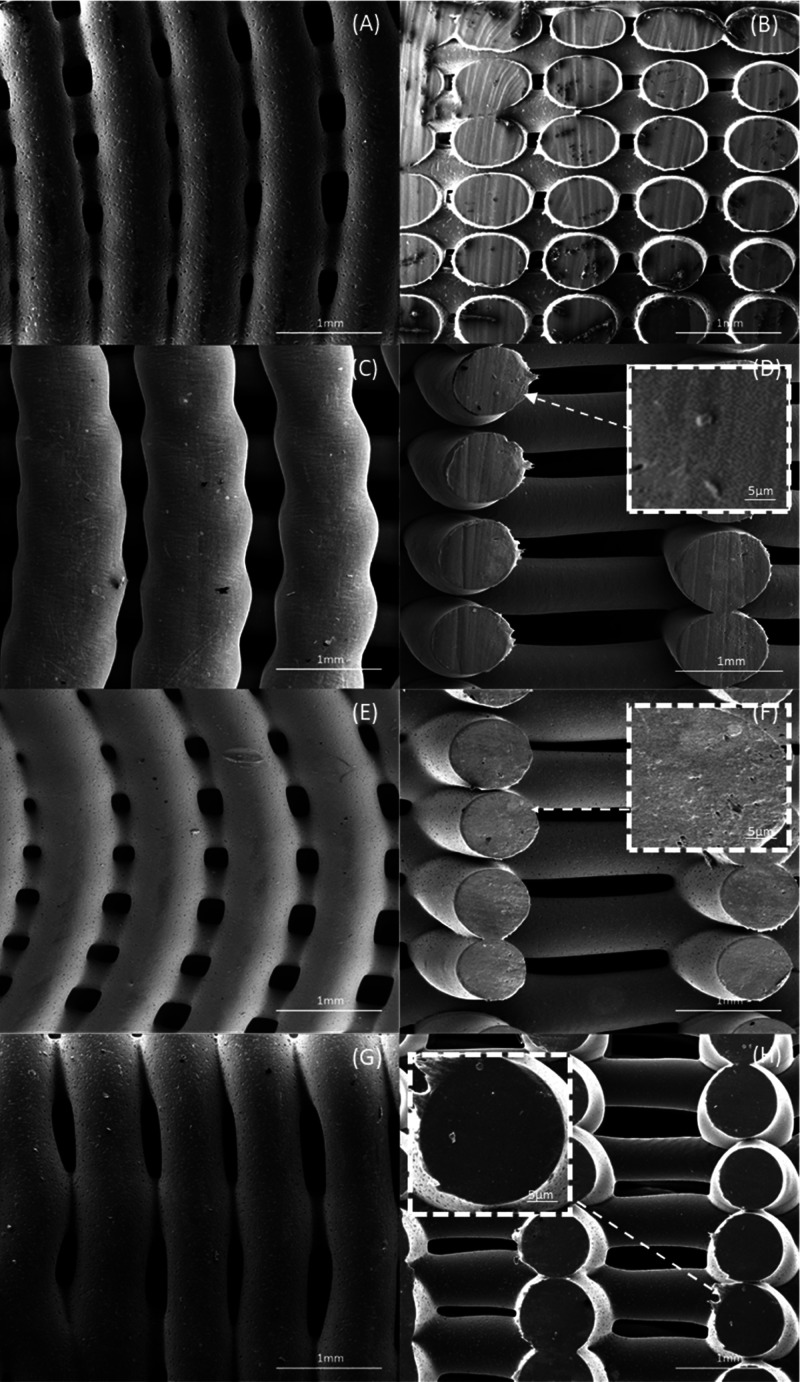
SEM images of bone bricks (top and cross-section views) (case 7) on (A, B) PCL bone brick, (C, D) HA 10 wt % bone brick, (E, F) HA 15 wt %, and (G, H) HA 20 wt % bone brick.

SEM images of the top view of 15 wt % HA and 15 wt % TCP bone bricks (case 5 and case 6) are presented in Figure 4, showing that the pore size decreases from the outer region to the internal region of the bone brick. Figures 5 and6 present SEM images of printed bone bricks with different material compositions (Case 7). Filament width values, considering the initial design value of 330 μm, are presented in Table S1 (Supporting Information). Results show some differences between the initially designed and acquired values, which can be attributed to the use of the same fabrication conditions despite the rheological differences of the considered blends. Moreover, results also show that by increasing the ceramic content the filament width decreases and consequently the pore size increases. This can be attributed to the higher viscosities of the PCL/HA and PCL/TCP blends, leading to a higher resistance flow, thus reducing the amount of extruded material as previously reported by our group.^38^ Therefore, for the same processing conditions, the amount of molten PCL/HA extruded is higher than the PCL/TCP, leading to high filament widths. The pore size decreases by increasing the number of double filaments (from 25 to 38), and a similar effect was observed by increasing the number of spiral filaments (from 6 to 14). Likewise, for the same reinforcement material, it was possible to observe that the filament width decreases by increasing the concentration of the ceramic material from 10 to 20 wt %, while the pore size slightly increases by increasing the ceramic concentration. Additionally, bone bricks containing pure PCL presented micropore structures on the filaments’ surface (Figure 5A), while bone bricks containing ceramic particles show less micropores (Figures 5C,E,G and6C,E,G). Smooth surface characteristics can be explained by the recrystallization process and the crystals size of the ceramic particles.^39^

**Figure 6:**
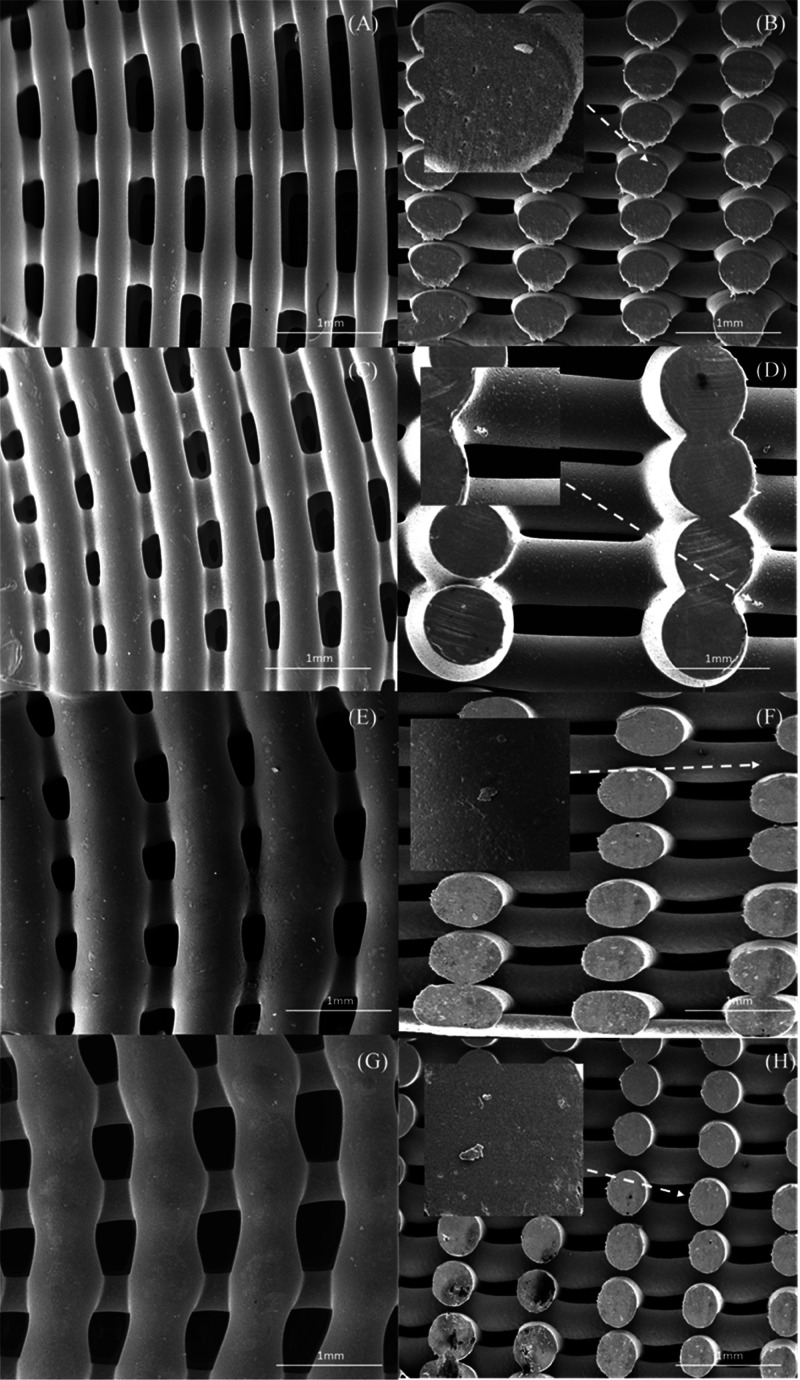
SEM images of bone bricks (top and cross-section views) (case 7) on (A, B) TCP 10 wt % bone brick, (C, D) TCP 15 wt % bone brick, (E, F) TCP 20 wt %, and (G, H) HA/TCP 10/10 wt % bone brick.

### 3.2 Thermal Gravimetric Analysis

**Figure 7:**
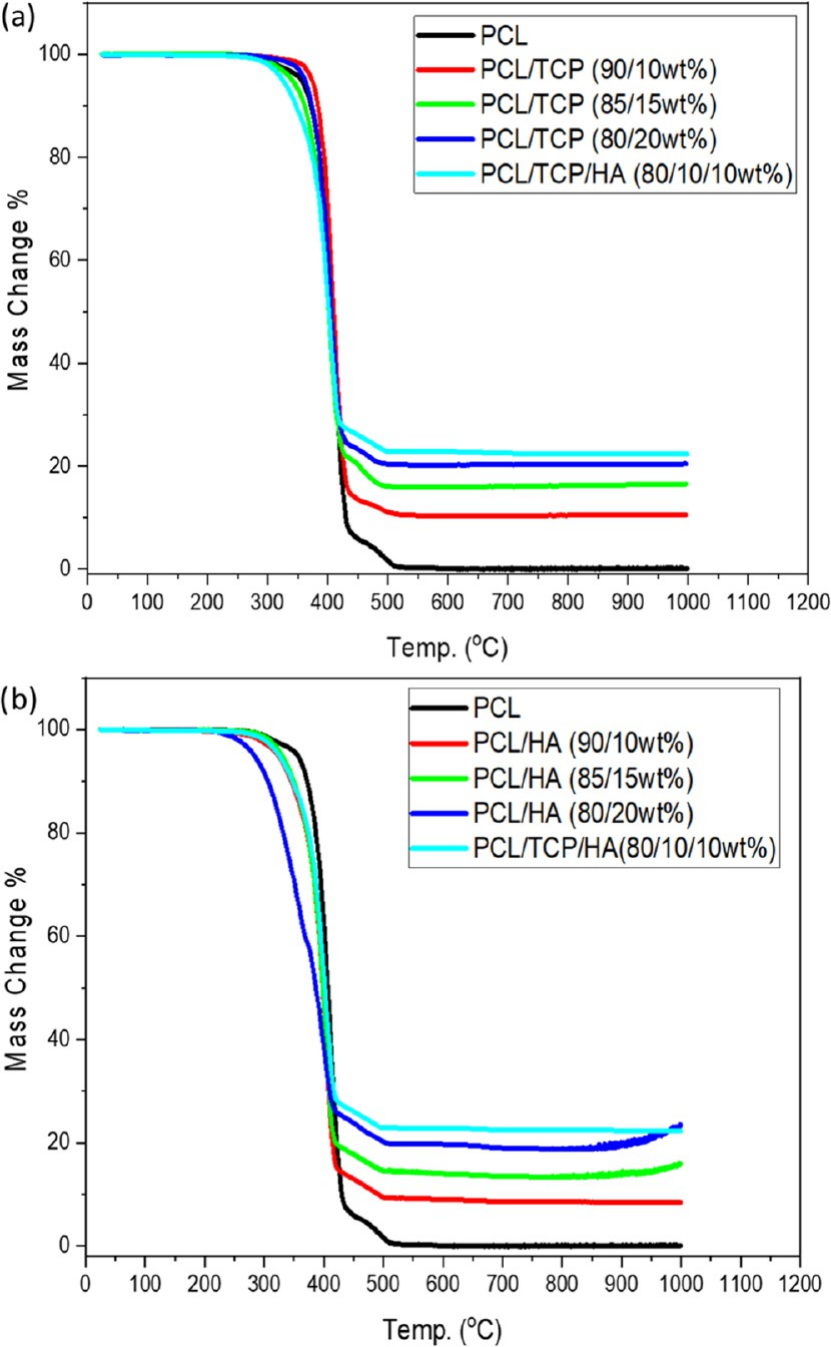
TGA curves of (a) TCP bone bricks and (b) HA bone bricks.

Thermal gravimetric analysis (TGA) results show that the bone bricks exhibit degradation temperatures ranging between 418 and 437 °C, after which only the inorganic materials remain (Figure 7). Moreover, results show that the degradation temperature decreases by increasing the ceramic content. In the case of PCL/HA bricks, it is possible to observe that the increase of HA seems to accelerate the onset of the degradation process. This can be attributed to the presence of the mineral phase in the matrix, which disrupted the formation of PCL crystallites, the size of HA particles (nm), and the distribution of HA particles in the PCL matrix.^40,41^ This effect is not evident in the case of PCL/TCP bone bricks probably due to the larger TCP particle size and eventually a homogeneous distribution of TCP particles in the PCL matrix, leading to more thermally stable bone bricks. Based on these results, and considering that the fabrication temperature is 90 °C, it is possible to conclude that the printing process does not induce any degradation. Moreover, the levels of HA and TCP determined by TGA (Table 1) suggest that the melt blending process is an effective method for the preparation of composite blends.

**Table 1:** Degradation Temperature of the Bone Bricks and the Corresponding Mineral Content (Comparison between Designed and Obtained Contents of HA and TCP on Bone Bricks)

parameters/material configuration	designed ceramic content (wt %)	measured ceramic content (wt %)	degradation temperature (°C)
PCL	0	0	437.11 ± 0.21
PCL/HA	10	9.49 ± 0.03	430 ± 0.3
PCL/HA	15	14.31 ± 0.18	425.8 ± 0.2
PCL/HA	20	20.27 ± 0.001	425.55 ± 0.38
PCL/TCP	10	10.63 ± 0.06	435.44 ± 0.18
PCL/TCP	15	16.09 ± 0.008	418.70 ± 0.31
PCL/TCP	20	20.38 ± 0.05	424.04 ± 0.23
PCL/TCP/HA	10/10	22.91 ± 0.007	432.15 ± 3.03

### 3.3 Chemical Analysis

**Figure 8:**
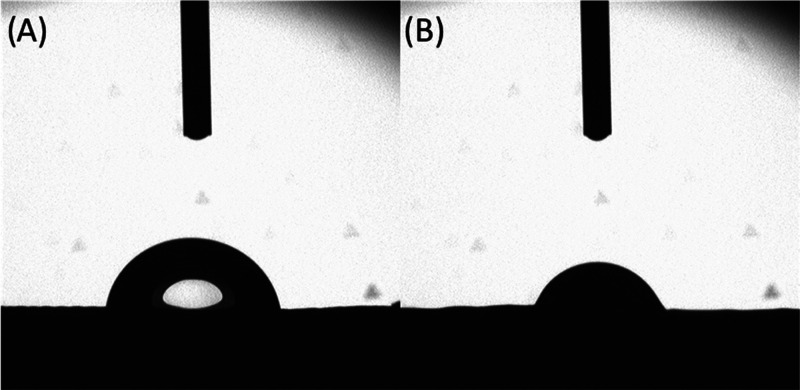
Water droplet on a PCL bone brick filament at 0 s (A) and 20 s (B).

Figure 8 illustrates the water droplet on a PCL bone brick at two different time points. Detailed results are presented as the Supporting Information (Table S2). As observed, the contact angle decreases with time, showing that the bone bricks are absorbing the water. Moreover, results show that by increasing the number of zig-zag and spiral layers, the contact angle decreases. This can be attributed to the increase of pore size from the inner regions to the outer regions. Additionally, by increasing the gradient of the bone bricks, and moving from the inner region to the outer region, the contact angle slightly decreases. Furthermore, results seem to indicate that the addition of ceramic particles has no effect on the bone bricks’ hydrophobicity.

**Figure 9:**
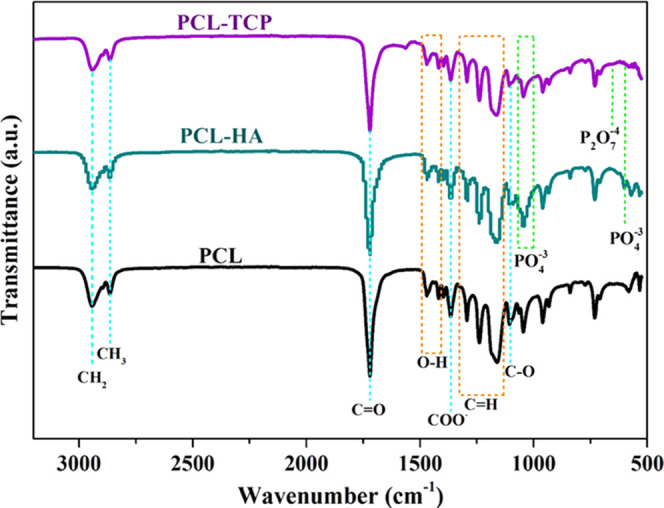
FTIR spectra of pure PCL and composite printed bone bricks.

FTIR, used to investigate the chemical composition of the printed nanocomposite fibers, confirmed the presence of ceramic particles within the bone bricks (Figure 9). As observed from the spectra, bonds of phosphate groups are recognizable in the PCL-HA and PCL-TCP samples. In the composite samples, the characteristic PO_4_^–3^ absorption bands attributed to HA and TCP particles are detected at 601 and 1041 cm^–1^. The characteristic peak of TCP associated with the P2O7-4 group (727–1200 cm^–1^) was also observed in the PCL-TCP spectrum. Compared to the PCL-HA spectrum, the intensity of the phosphate peak in the PCL-TCP was reduced. As previously reported, this might occur due to the water absorption of O–H groups from the environment.^40,41^ In all cases, it was possible to observe the presence of relevant PCL peaks: CH_2_ peaks at 2942 and 2863 cm^–1^, C=O at 1724 cm^–1^, COO^–^ at 1364 cm^–1^, and C–O at 1103 cm^–1^. These results indicate that there are no chemical transformations during both the material preparation and the fabrication stages, confirming the presence of bioceramic particles on the surface of printed filaments.

**Figure 10:**
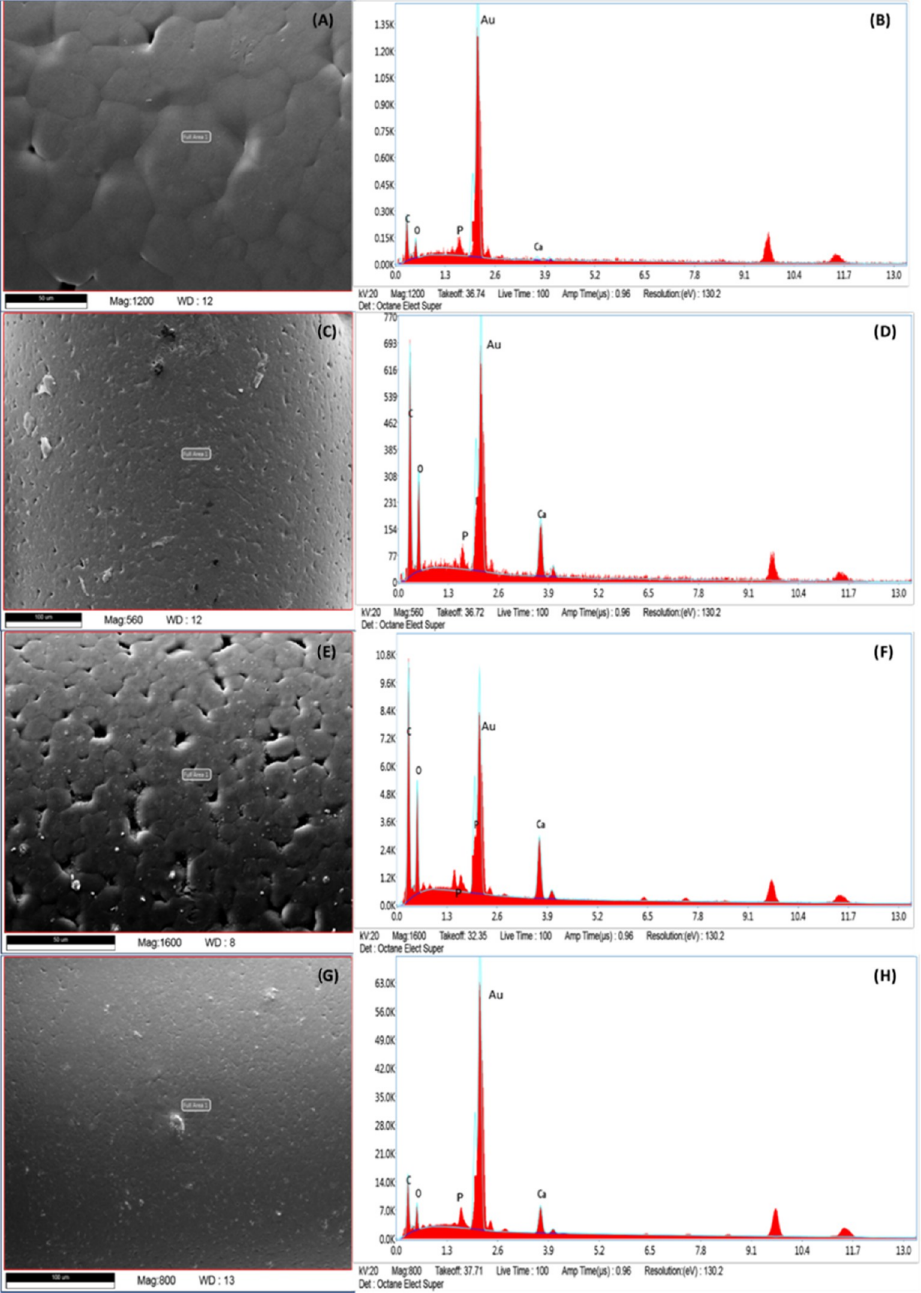
SEM and EDX spectra of PCL bone brick (A, B), PCL/TCP (80/20 wt %) (C, D), PCL/HA (80/20 wt %) (E, F), and PCL/HA/TCP (80/10/10 wt %) (G, H).

Chemical composition analysis was carried out using EDX spectroscopy to calculate the percentage (wt %) of carbon (C), calcium (Ca), oxygen (O), and phosphate (P) on the bone bricks (Figure 10). Table 2 shows the element concentration on the surface of the bricks. As observed, bone bricks containing ceramic reinforcements showed less C content but higher Ca, O, and P contents compared to PCL bone bricks. Results also show that by increasing the ceramic content the Ca, O, and P contents also increase. For a similar amount of ceramic content, the levels of Ca, O, and P are higher in the case of PCL/HA bone bricks.

**Table 2:** Element Composition of Bone Bricks for Different Materials

material composition/ element composition (wt %)	PCL	PCL/HA/TCP (80/10/10 wt %)	PCL/TCP (90/10 wt %)	PCL/TCP (85/15 wt %)	PCL/TCP (80/20 wt %)	PCL/HA (90/10 wt %)	PCL/HA (85/15 wt %)	PCL/HA (80/20 wt %)
C (carbon)	77.65	68.93	71.76	66.39	61.7	70.31	63.3	60.1
Ca (calcium)	0	5.41	3.8	6.89	9.2	4.2	8.8	9.9
O (oxygen)	22.35	23.41	22.54	23.92	24.73	22.8	24.7	25.2
P (phosphate)	0	2.25	1.9	2.8	4.73	2.69	3.2	4.8

### 3.4 Physical Analysis

**Figure 11:**
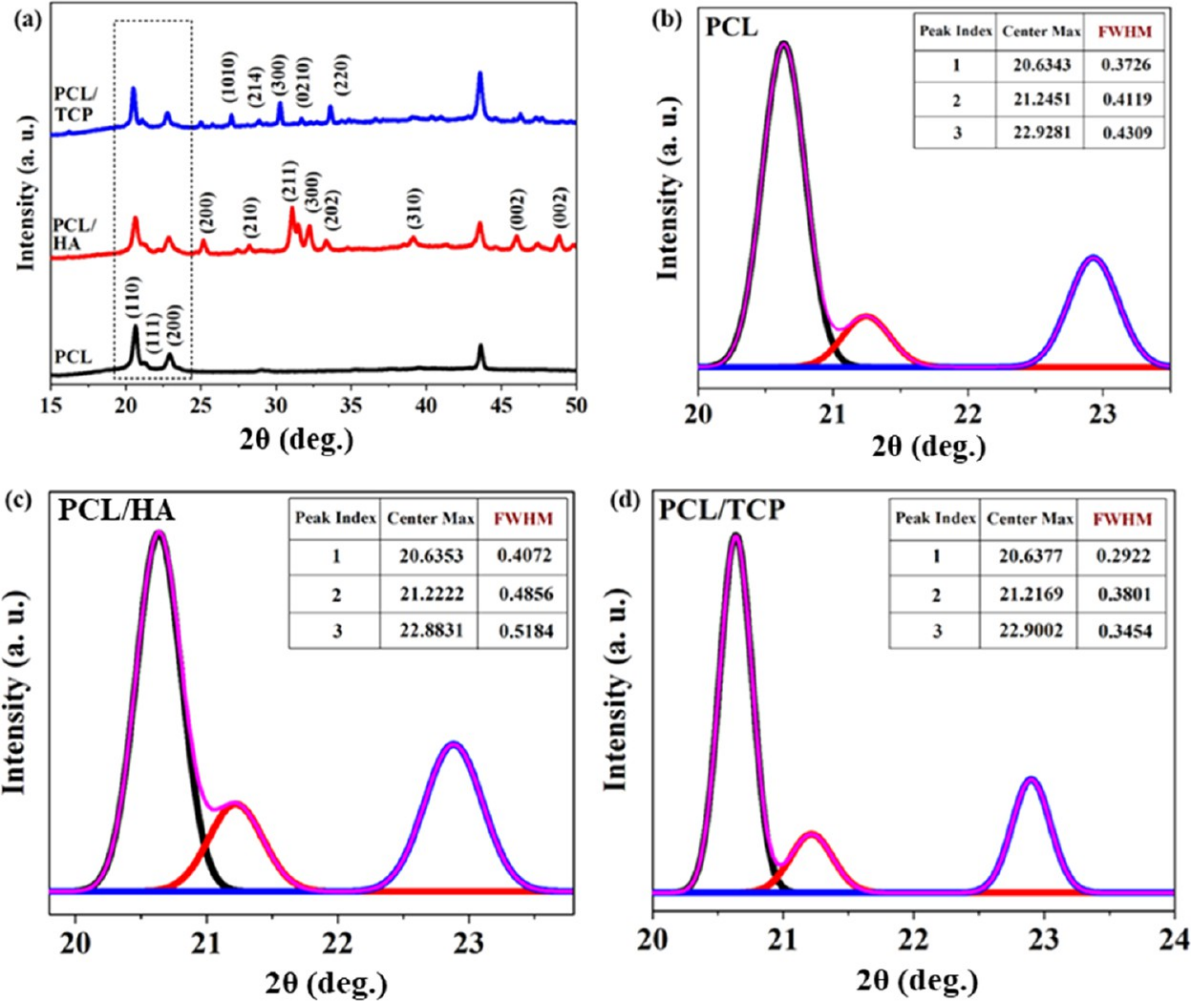
High-resolution XRD patterns for (a) PCL, PCL/HA, and PCL/TCP bone bricks; (b) PCL bone bricks; (c) PCL/HA bone bricks; and (d) PCL/TCP bone bricks in the range of 2θ = 20–24°.

The XRD pattern confirms the crystalline phase of PCL, in the composite bone bricks (HA and TCP) with 2θ ranging from 20 to 24° (Figure 11). The characteristic peaks reflecting (110), (111), and (200) crystal planes are found in all bone brick samples. Moreover, the HA and TCP peaks also match well with the TCP and HA standard JCPD cards (09-0169 and 09-0432, respectively). These results also confirm that the materials did not experience any chemical transformation. The effects of TCP and HA particles on PCL crystallization were also investigated. As observed from Figure 11b–d and Table 3, the full width at half-maximum (FWHM) amounts of PCL/HA increase, while those of PCL/TCP decrease in comparison to PCL samples. According to the Scherrer equation, the crystallinity and crystalline size decrease by increasing the FWHM value.^42^ Therefore, results show that, for the same processing conditions, the incorporation of HA in the PCL matrix induces lower crystallinity than the incorporation of TCP. Moreover, the addition of HA and TCP particles reduces the overall percentage of the PCL crystal structure in the composite samples. As presented in Table 3, PCL/TCP filaments exhibit larger crystalline size structures than both PCL/HA and PCL samples. This might also be attributed to the better compatibility of TCP in the PCL matrix, allowing better polymer chain mobility to disentangle, reorient, and increase the crystalline size.^35^

**Table 3:** Calculated PCL Crystallinity in the Bone Bricks and Their Crystalline Size^a^

samples	PCL crystallinity (%)	FWHM (°)	crystalline size (nm)
PCL	68.9	0.3726	21.7
PCL/HA	22.1	0.4072	19.8
PCL/TCP	26.8	0.2922	27.6

a The highest peak (crystal plane 110) shown in Figure 9 was used to calculate the crystallinity and crystalline size.

**Figure 12:**
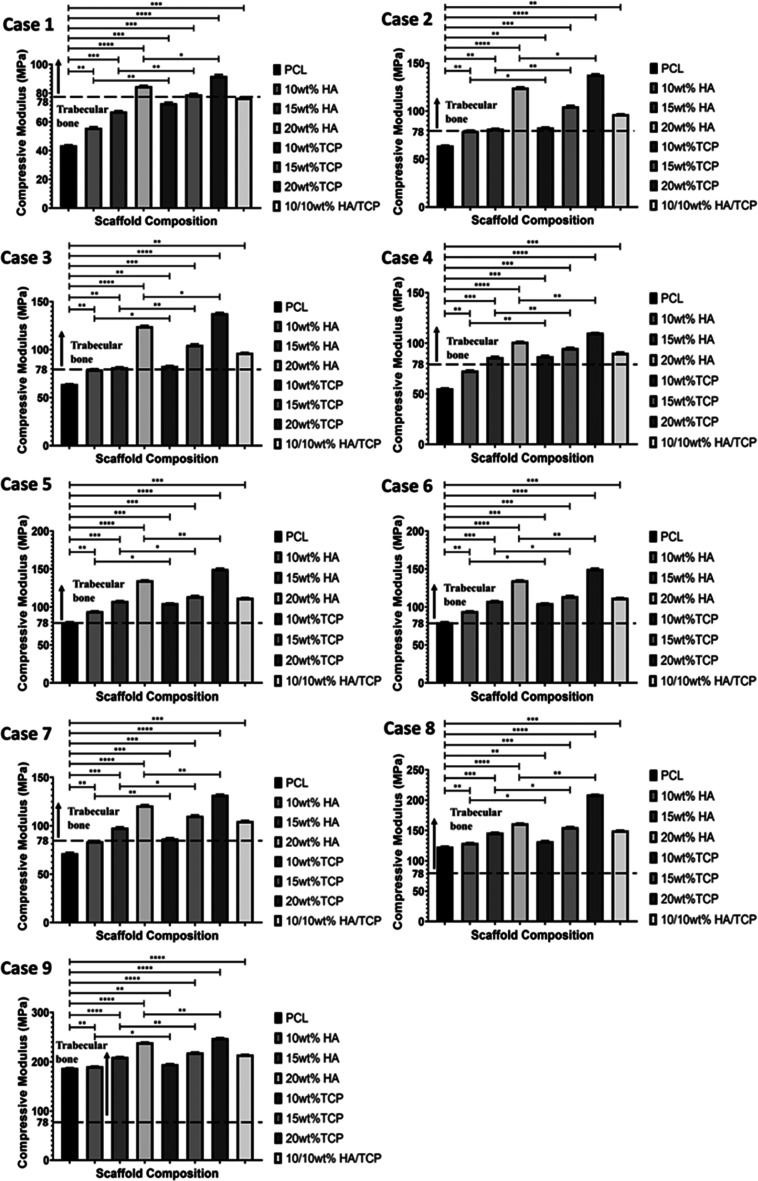
Compressive modulus as a function of bone brick architecture and material composition. *Statistical evidence (*p* < 0.05) analyzed by one-way ANOVA and Tukey’s post-test. * indicates statistical evidence (*p* < 0.05); **, *** illustrate the differences between the compression results.

As shown in Figure 12 and Table 4, the mechanical behavior of the fabricated bone bricks strongly depends on their architecture and material composition. Results show that bone bricks containing TCP particles exhibit higher compressive modulus and yield strength values in comparison to PCL/HA and PCL/HA/TCP. Moreover, for the same bone brick architecture, compressive modulus and yield strength increase by increasing the inorganic material content. This can be attributed to the higher crystallinity and crystal sizes of PCL/TCP filaments (Table 3). For the same ceramic content and double zig-zag filaments, compressive modulus and yield strength increase by increasing the spiral filaments. Moreover, results showed that by increasing the number of double zig-zag filaments, bone bricks with a similar material composition and level of spiral filaments present a higher compressive modulus and yield strength, which can be related to the reduction on the overall porosity. Additionally, results show that bone bricks can reach compressive modulus values in the region of the trabecular bone and present a higher compressive modulus than regular shape scaffolds with similar material compositions.^37,43−45^ Results obtained from square-shaped scaffolds, of 300 μm pore size, showed compressive modulus values of 48–75 MPa (80/20 wt % PCL/HA scaffolds) and 88 MPa (80/20 wt % PCL/TCP scaffolds).^34^ Other studies considering square- or circular-shaped scaffolds with regular pore shapes and pore sizes produced using different path planning strategies (0/90, 0/45/90, or 0/30/60/120 lay-dawn strategies) also showed that the addition of ceramic particles into the polymeric material increases the mechanical properties of produced scaffolds but not in the same order of magnitude as bone.^37,46−52^ However, in this study, it was possible to achieve a compressive modulus of 248 ± 0.7 MPa and a yield strength of 9.36 ± 0.34 MPa (case 9).

**Table 4:** 0.2% Offset Yield Strength Results of Bone Bricks with Different Architectures and Material Compositions

material composition/ configuration	PCL	PCL/HA/TCP (80/10/10 wt %)	PCL/ TCP (90/10 wt %)	PCL/TCP (85/15 wt %)	PCL/ TCP (80/20 wt %)	PCL/HA (90/10 wt %)	PCL/HA (85/15 wt %)	PCL/HA (80/20 wt %)
case 1	2.27 ± 0.5	2.68 ± 0.5	2.59 ± 0.6	2.75 ± 0.5	3.76 ± 0.7	2.45 ± 0.4	2.66 ± 0.2	3.26 ± 0.4
case 2	2.49 ± 0.4	3.67 ± 0.2	3.31 ± 0.4	3.68 ± 0.6	4.58 ± 0.1	2.93 ± 0.4	3.63 ± 0.3	3.77 ± 0.3
case 3	2.79 ± 0.1	4.12 ± 0.2	3.9 ± 0.5	4.18 ± 0.5	4.93 ± 0.6	3.42 ± 0.6	4.05 ± 0.1	4.68 ± 0.3
case 4	2.74 ± 0.8	3.94 ± 0.2	3.13 ± 0.3	4.05 ± 0.5	4.28 ± 0.3	2.97 ± 0.5	3.86 ± 0.3	4.15 ± 0.3
case 5	3.53 ± 1	4.5 ± 0.3	4.16 ± 0.7	4.55 ± 0.3	4.79 ± 0.3	3.87 ± 0.1	4.43 ± 0.9	4.7 ± 0.5
case 6	4.4 ± 0.8	4.9 ± 0.6	4.73 ± 0.5	4.95 ± 0.4	5.58 ± 0.1	4.57 ± 0.4	4.8 ± 0.4	5.24 ± 0.8
case 7	5.81 ± 0.9	5.4 ± 0.4	5.3 ± 0.3	5.45 ± 0.6	5.98 ± 0.7	5.12 ± 0.7	5.35 ± 0.5	5.78 ± 0.4
case 8	6.72 ± 1	7.1 ± 0.3	6.99 ± 0.2	7.1 ± 0.4	7.39 ± 0.2	6.85 ± 0.6	7.02 ± 0.3	7.23 ± 0.2
case 9	7.5 ± 0.3	8.5 ± 0.4	7.82 ± 0.2	8.58 ± 0.5	9.36 ± 0.3	7.69 ± 0.7	8.31 ± 0.2	8.97 ± 0.4

### 3.5 Biological Analysis

**Figure 13:**
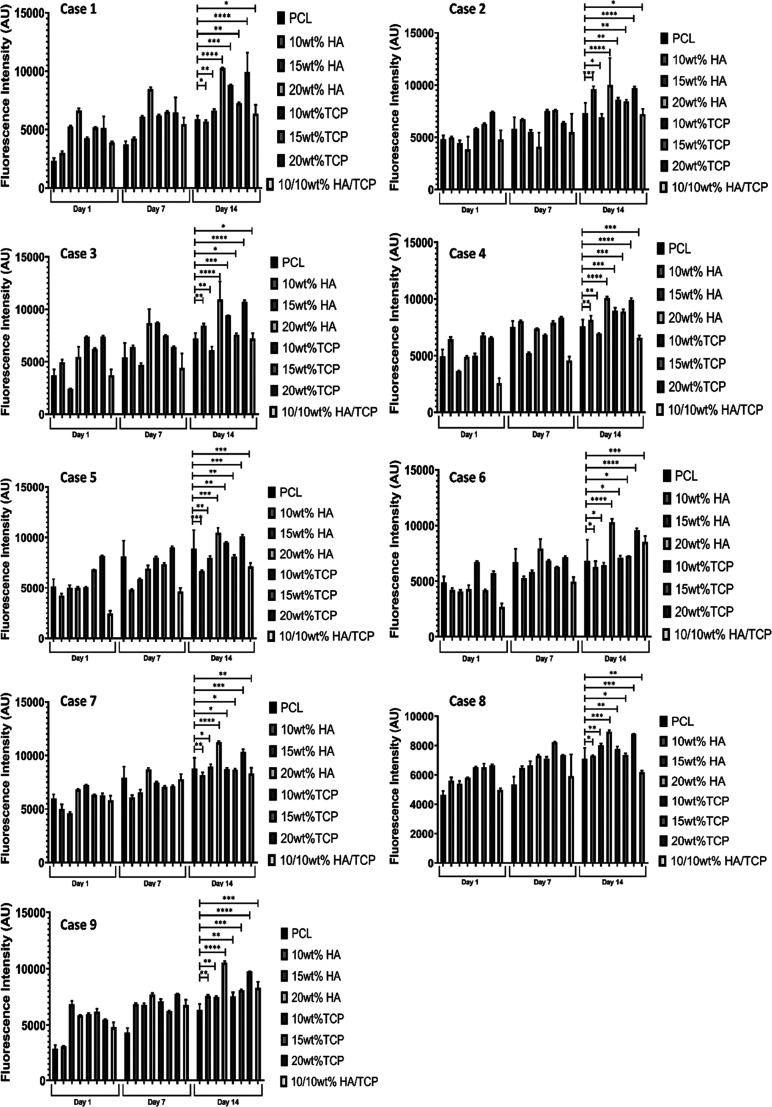
Average fluorescence intensity as a function of bone brick architecture and material composition for different days after cell seeding. *Statistical evidence (*p* < 0.05) analyzed by one-way ANOVA and Tukey’s post-test. * indicates statistical evidence (*p* < 0.05); **, *** illustrate the differences between the compression results.

Figure 13 presents the fluorescence intensity as a function of material composition and bone brick architecture on different days (days 1, 7, and 14) after cell seeding. As observed, bone bricks do not present any cytotoxicity effects, being able to sustain cell attachment and proliferation. Results show that, for the same architecture of a ceramic content, HA bone bricks exhibit a higher metabolic activity than TCP and PCL bone bricks. The highest fluorescence intensity values were observed for HA bone bricks (20 wt %) and case 7 (38 double filaments and 6 spiral filaments). Moreover, the overall fluorescence intensity increases from case 1 (larger pore size) to case 9 (smaller pore size). Case 1 shows low cell proliferation and bridging between adjacent filaments as it presents higher average pore sizes, ranging from 741 μm in the case of PCL bricks to 800 μm in the case of bone bricks containing 20 wt % HA. On the contrary, case 7 exhibits the highest level of metabolic activity in comparison to the other cases due to its higher surface. Overall, cell metabolic activity increases by increasing the ceramic content. No statistically significant differences were observed between PCL/HA and PCL/TCP.

**Figure 14:**
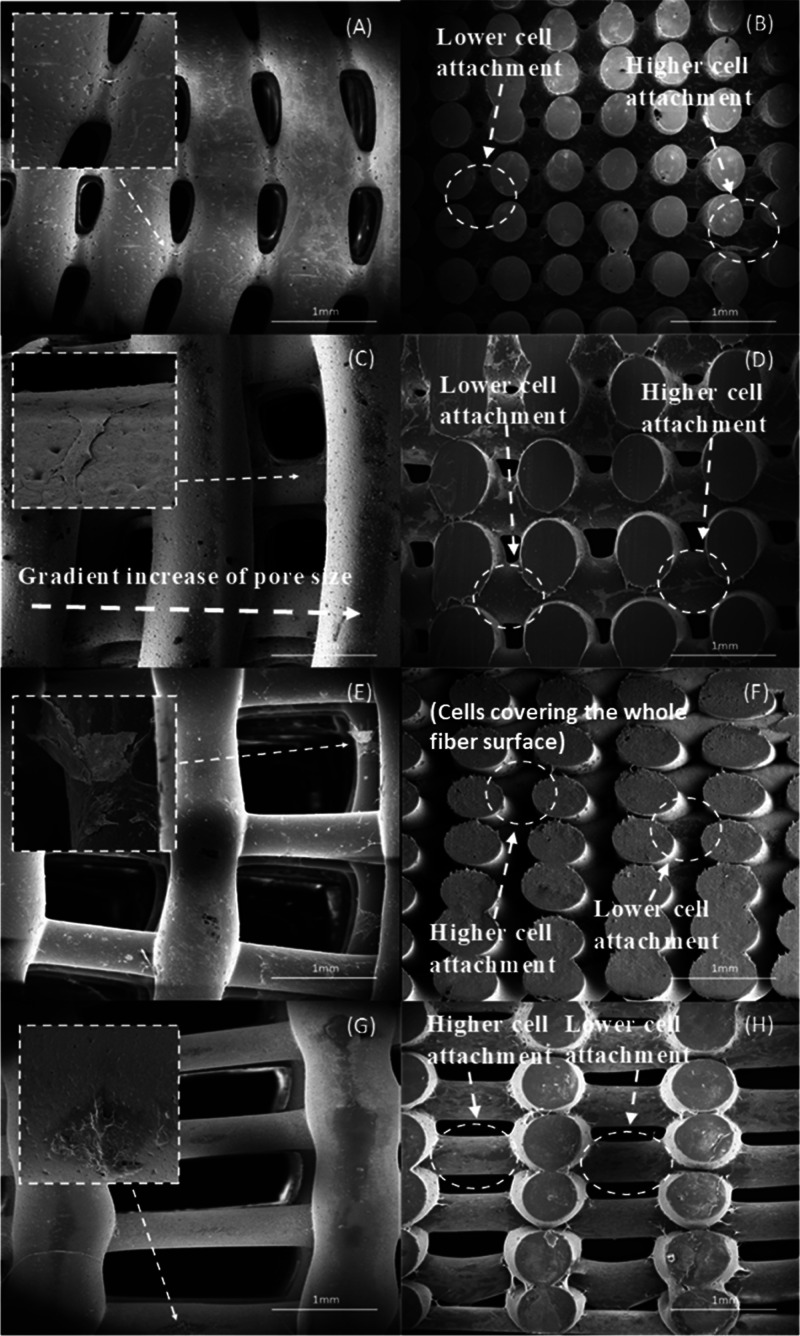
Top and cross-section SEM images showing cell spreading on bone bricks (case 7) with different material compositions for (A, B) PCL, (C, D) 10 wt % HA, (E, F) 15 wt % HA, and (G, H) 20 wt % HA.

**Figure 15:**
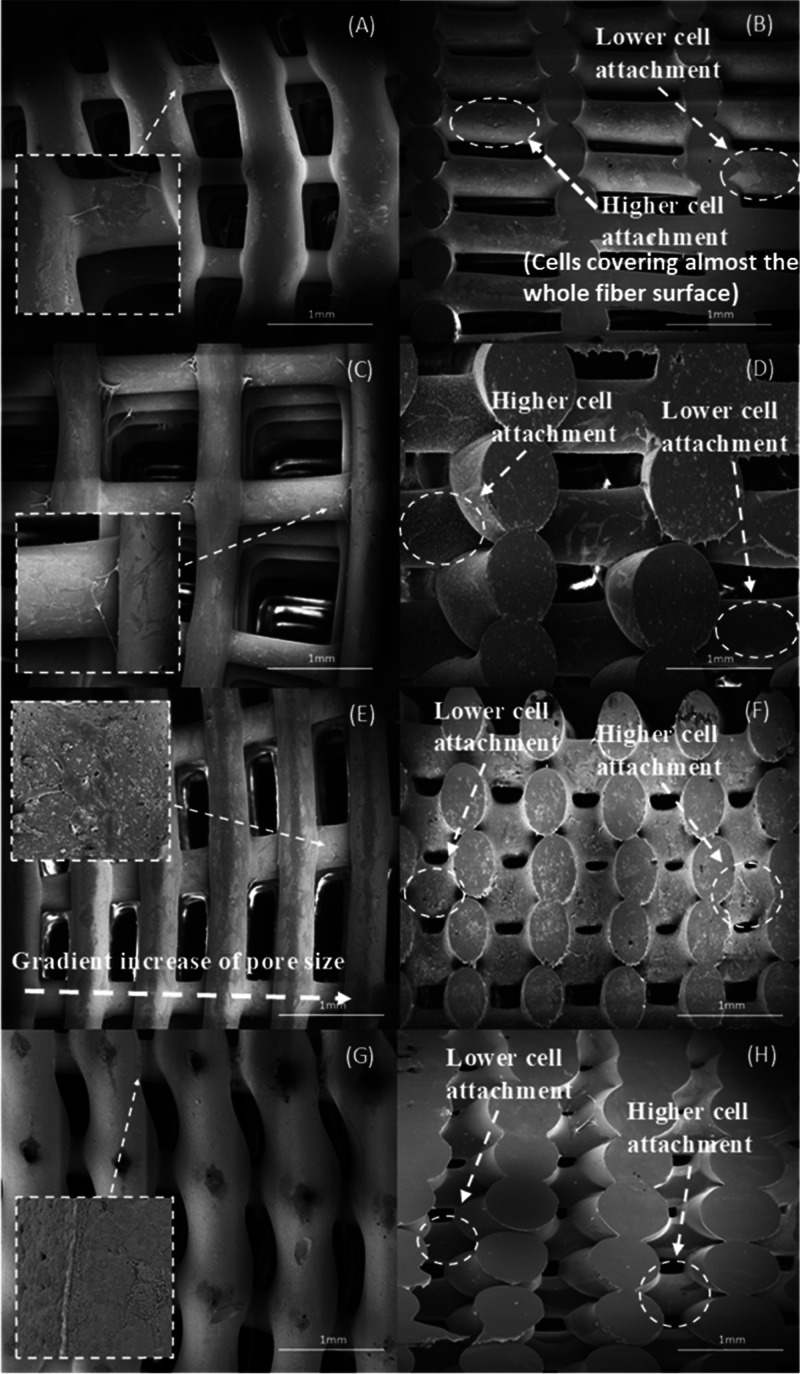
SEM images showing cell spreading on bone bricks (case 7) (top and cross section) for (A, B) 10 wt % TCP, (C, D) 15 wt % TCP, (E, F) 20 wt % TCP, and (G, H) 10/10 wt % HA/TCP.

**Figure 16:**
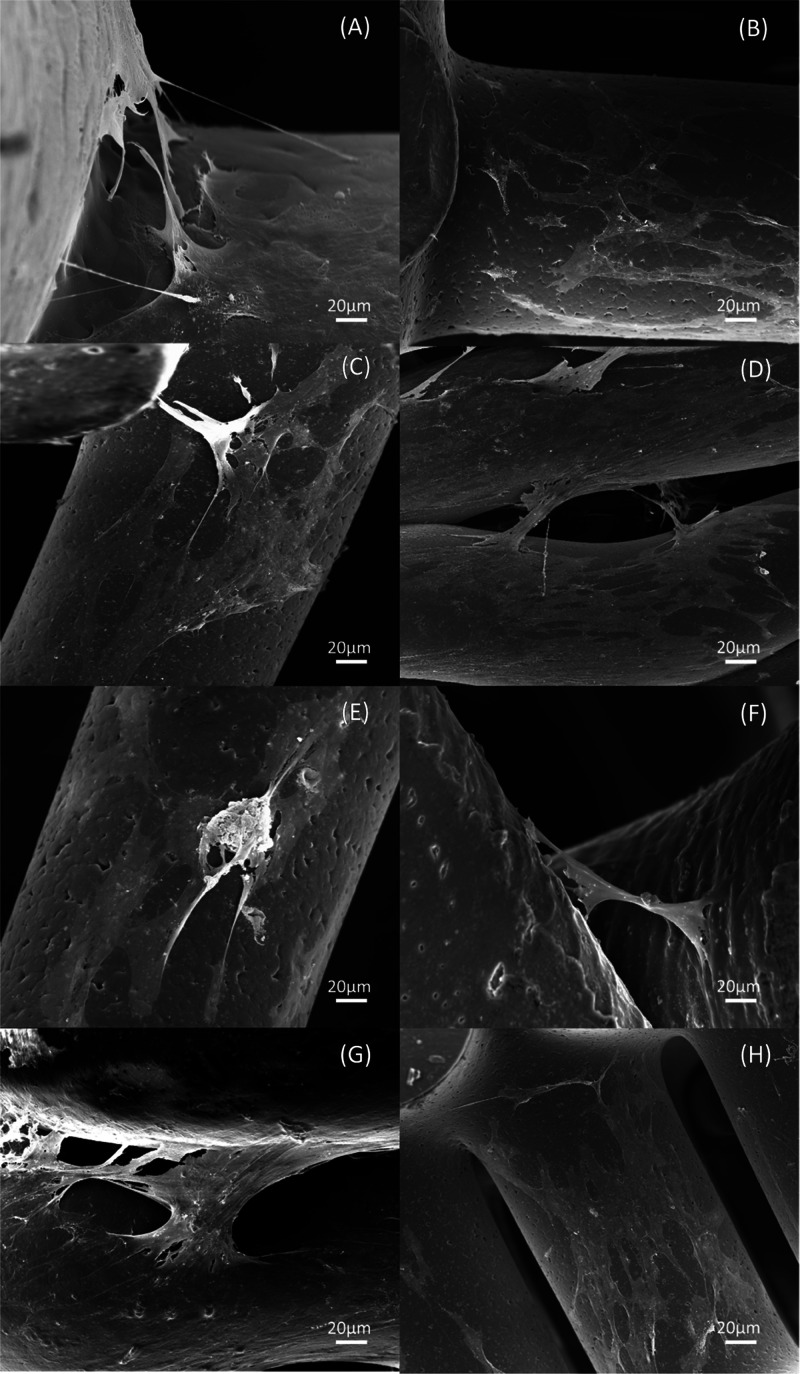
SEM images of cells attached and spreading on bone bricks (case 7) as a function of material composition for (A) PCL bone brick, (B) 10 wt % HA bone brick, (C) 15 wt % HA bone brick, (D) 20 wt % HA bone brick, (E) 10 wt % TCP bone brick, (F) 15 wt % TCP bone brick, (G) 15 wt % TCP bone brick, and (H) 10/10 wt % HA/TCP bone brick.

SEM images of bone bricks (case 7) (Figures 14 and 15) after 14 days of cell seeding present cells spreading on the surface of bone bricks (Figures 14A,C,E,G and15A,C,E,G). Moreover, it can be observed that by reducing the pore size of the bone bricks, the number of cells increases. Results also seem to indicate the presence of more cells in the outer regions than in the inner regions of the bricks (Figures 14B,D,F,H and15B,D,F,H). It is also possible to observe that cells are creating bridges mainly on adjacent layers with small pore sizes. Figure 16 presents cells attached and spreading on the surface of bone bricks (case 7).

**Figure 17:**
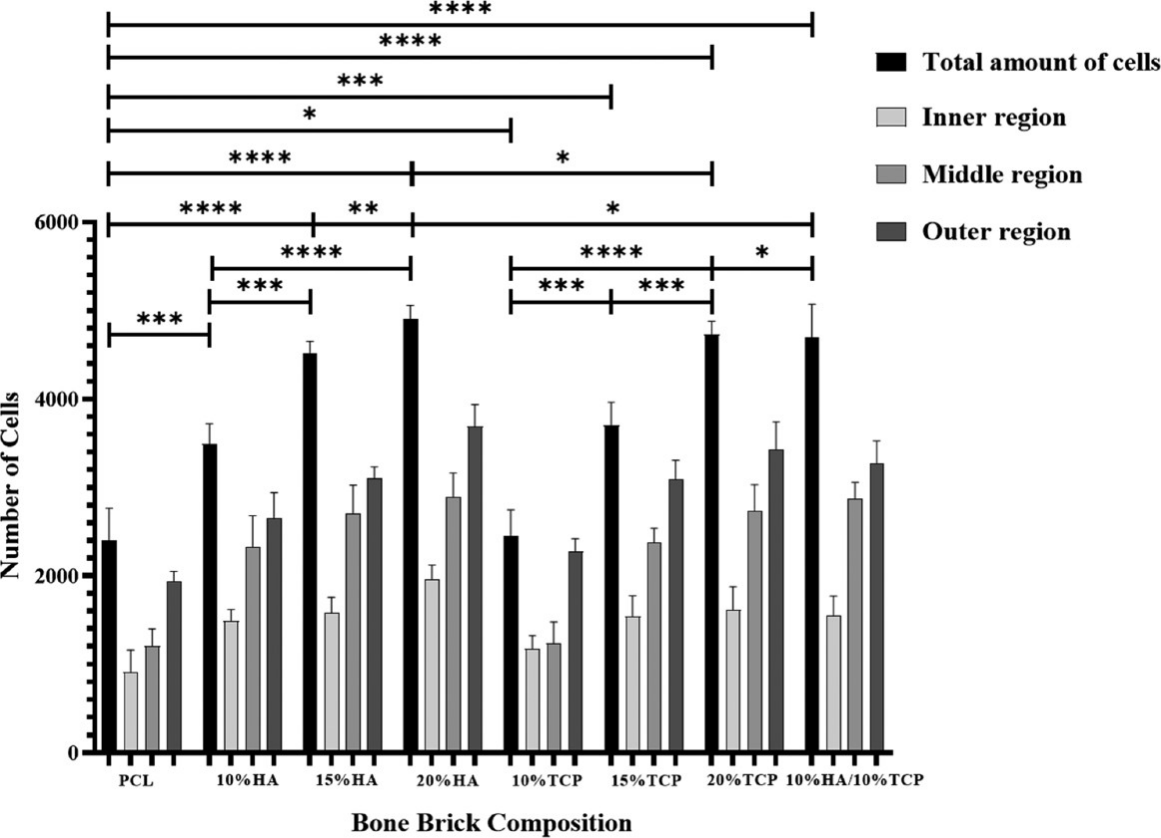
Number of cells at different regions on the bone bricks (case 7). For clarity, statistical analysis was conducted considering only the total number of cells for the different material compositions.

The cell number was quantified using confocal images and ImageJ software. Figure 17 shows the number of cells at different regions of bone bricks (inner, middle, and outer regions). As observed, more cells are found at the outer regions, while the number of cells is lower at the inner region, as larger pore sizes facilitate the supply of nutrients and oxygen.

**Figure 18:**
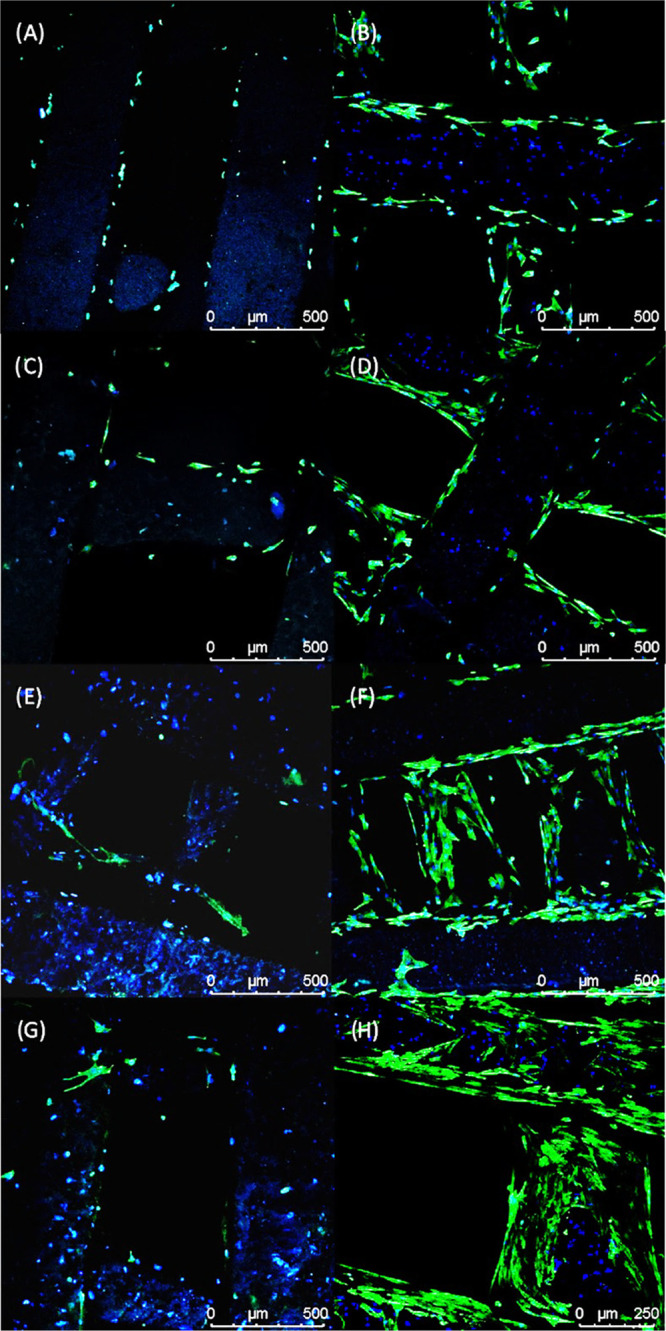
Confocal images of cells attached and spreading on day 1 (A, C, E, G) and day 14 (B, D, F, H) (case 7) on (A, B) PCL bone brick, (C, D) 10 wt % HA bone brick, (E, F) 15 wt % HA bone brick, and (G, H) 20 wt % HA bone brick.

Figures 18 and19 show the confocal images of cells on bone bricks (case 7) on day 1 and day 14. On day 1, confocal images show a low number of cells attached to the bone bricks. At this time point, it is also possible to observe the presence of more cells attached to PCL/HA bricks than to PCL and PCL/TCP bricks. From day 1 to day 14, the number of cells significantly increases, spreading over the printed fibers and bridging the pores.

**Figure 19:**
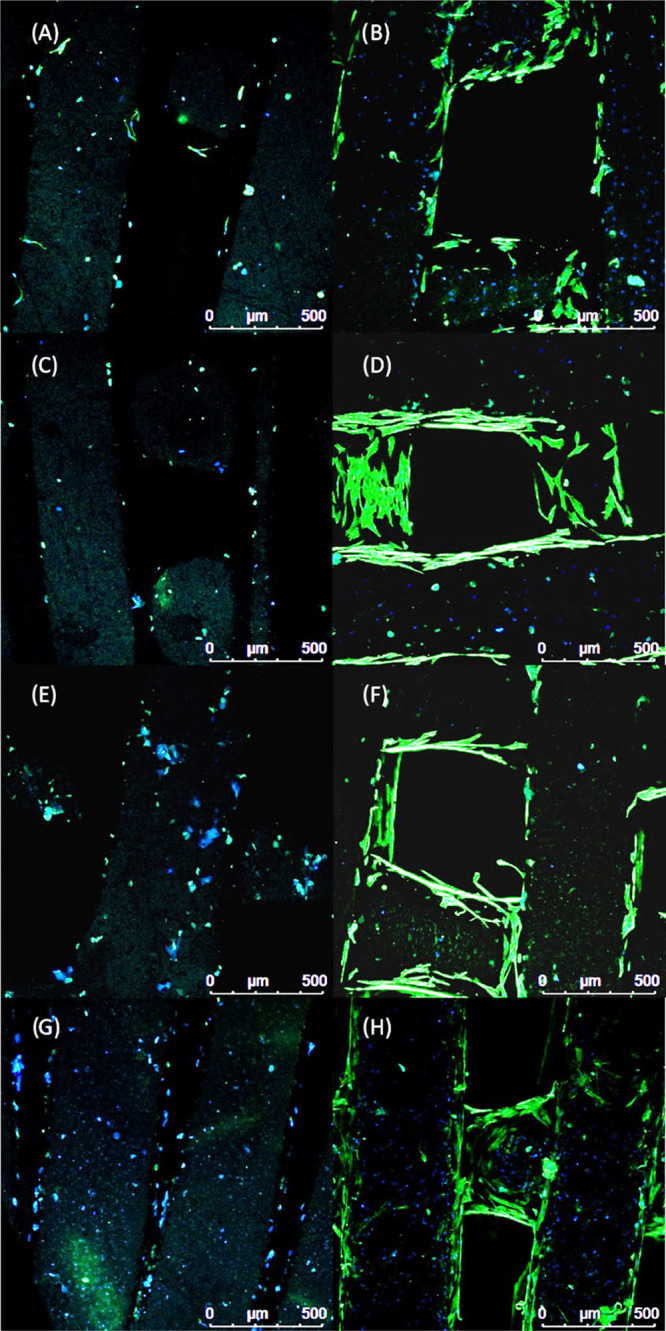
Confocal images of cells attached and spreading on day 1 (A, C, E, G) and day 14 (B, D, F, H) on bone bricks (case 7) presenting different material compositions. (A, B) Correspond to 10/10 wt % HA/TCP bone bricks; (C, D) correspond to 10 wt % TCP bone bricks; (E, F) correspond to 15 wt % TCP bone bricks; and (G, H) correspond to 20 wt % TCP bone bricks.

## 4 Conclusions

This paper investigates the effects of the geometrical architecture and material composition on the morphological, thermal, chemical, physical, and biological characteristics of novel anatomically designed scaffolds. Based on data previously obtained from anthropometric measurements, bone brick structures with nonuniform pore sizes were designed and produced using 3D printing. A novel tool-path algorithm allowed one to obtained bone bricks with different numbers of double zig-zag and spiral filaments, thus enabling one to control the overall porosity and pore size gradient, maximizing both mechanical and biological performance.

Different PCL/ceramic blends were prepared using a melt blending approach, which is a simple and effective approach as observed by TGA. Results also show that the considered processing conditions do not induce any chemical transformations. Contact angle tests show that the addition of ceramic materials has no impact on the hydrophilicity of the bone bricks, while XRD results show that the addition of TCP particles leads to the formation of larger PCL crystalline structures and high crystallinity values in comparison to the addition of HA particles. This partially explains the better mechanical properties observed for the PCL/TCP bone bricks in comparison to the other investigated bone bricks. However, bone bricks containing higher concentrations of HA particles exhibit better cell attachment and spreading. Results also show that both the mechanical and biological properties are highly dependent on the architecture of bone bricks. Table 5 summarizes the obtained morphological, mechanical, and biological results obtained for bone bricks. As observed, TCP-contained bone bricks present the highest mechanical properties (compression modulus value of 248 MPa and yield strength value of 9.36 MPa), while HA bone bricks exhibit the highest cell metabolic activity (11216 AU). Based on both mechanical and biological properties of all considered bone bricks, results seem to indicate that case 9 corresponds to the most suitable bone bricks. Case 9 corresponds to noncytotoxic bone bricks able to support cell attachment and proliferation and presenting mechanical properties suitable for bone tissue engineering applications.

**Table 5:** Results of (a) Mechanical, (b) Biological, and (c) Pore Size Characteristics for All Bone Bricks

material composition (wt %)	design	(a)	(b)	(c)
PCL	case 1	43.55	5871.05	741
	case 2	64	7298.04	652
	case 3	94.59	7121.33	465
	case 4	55.74	7566.03	647
	case 5	79.65	8894.24	631
	case 6	115.78	6826.78	420
	case 7	71.6	8759.72	562
	case 8	123.23	7114.19	448
	case 9	187.43	6359.14	333
PCL/TCP (90/10 wt %)	case 1	72.88	8806.58	774
	case 2	82.8	8628.96	655
	case 3	125.66	9410.93	475
	case 4	87.11	8964.17	653
	case 5	104.69	9476.96	640
	case 6	146.38	7092.18	425
	case 7	86.72	8722.95	568
	case 8	132.08	7755.83	454
	case 9	195.33	7523.31	335
PCL/TCP (85/15 wt %)	case 1	79	7254.02	786
	case 2	104.91	8429.83	711
	case 3	135.85	7578.98	488
	case 4	95.14	8885.3	664
	case 5	114.05	8069.66	653
	case 6	161.08	7244.95	428
	case 7	110.36	8683.33	581
	case 8	155.32	7353.71	464
	case 9	218.71	8102.02	339
PCL/TCP (80/20 wt %)	case 1	91.98	9910.68	798
	case 2	137.8	9690.84	751
	case 3	160.2	10690.84	498
	case 4	110.29	9896.43	690
	case 5	149.98	10112.12	665
	case 6	188.58	9564.74	431
	case 7	131.88	10341.54	601
	case 8	208.95	8792.08	558
	case 9	247.99	9754.02	357
PCL/HA (90/10 wt %)	case 1	55.84	5671.93	784
	case 2	79.36	9609.02	659
	case 3	114.9	8424.4	481
	case 4	72.83	8151.22	682
	case 5	94.37	6666.24	649
	case 6	127.02	6265.93	435
	case 7	83.94	8149.93	575
	case 8	129.33	7290.79	450
	case 9	190.8	7538.64	331
PCL/HA (85/15 wt %)	case 1	67.21	6618.34	790
	case 2	81.6	6923.88	668
	case 3	131.45	6109.53	490
	case 4	86.2	6935.27	694
	case 5	107.73	7959.61	659
	case 6	155.12	6435.01	437
	case 7	98.02	8964.27	590
	case 8	146.1	8007.52	471
	case 9	209.74	7502.6	342
PCL/HA (80/20 wt %)	case 1	84.85	10262.82	800
	case 2	124.36	10012.18	754
	case 3	153.3	10929.01	504
	case 4	101.32	10091.15	704
	case 5	135.03	10454.43	668
	case 6	179.47	11286.12	434
	case 7	120.97	11216.73	613
	case 8	161.56	8940.97	564
	case 9	239.23	10531.73	373
PCL/HA/TCP (80/10/10 wt %)	case 1	76.8	6366.91	789
	case 2	96.7	7212.32	726
	case 3	132.47	7213.33	490
	case 4	90.4	6574.58	684
	case 5	111.98	7141.64	657
	case 6	157.1	8533.92	430
	case 7	104.97	8302.7	595
	case 8	149.8	6187.99	509
	case 9	214.61	8302.7	352

Based on this preliminary design study, bone bricks corresponding to case 9 will be further investigated both in vitro and in vivo. In vitro studies will focus on more complex mechanical studies considering static (tensile, torsion, bending, and combination of loads) and dynamic tests and the ability of these bone bricks to sustain osteogenic differentiation and mineralization and the effect of the pore size gradient on new bone formation. Finally, in vivo studies will be conducted using an ovine large-animal model to evaluate bone formation over time in the implant. A critical-sized bone defect measuring 6 cm will be created in the mid-diaphysis of the tibia in 10 sheep (5 control and 5 implant). The tibia will be stabilized by an external fixator with six hydroxyapatite-coated fixator pins: three above and three below the defect. The in-life period will be for 26 weeks. The control group will have external fixation, and the implant group will test the bone bricks with the external fixator. During the in-life period, the animals will be CT-scanned and radiographed at 6 and 12 weeks and will be given fluorescein bone markers to measure bone apposition between 12, 15, and 18 weeks. At termination, the animals will again be CT-scanned and radiographed. The bone mineral density in the defect site will be measured by pqCT. Undecalcified longitudinal histological sections will be made through the defect site. The location and the distance between the bone markers will be measured. The sections will be stained, and the amount of bone, soft tissue, and remaining implant will be quantitatively assessed using histomorphometry. The CTs and radiographs taken during the 6 month in-life phase and at termination will be used to quantitatively assess bone formation.

## Supporting Information Available

Morphological characteristics of bone brick structures for different configurations, showing the obtained pore size and filament width values for all considered bone bricks (Table S1); WCA results of bone brick structures at 0 and 20 s, which present the WCA values at different regions (inner, middle, and outer regions) of bone bricks (Table S2) (PDF)
