# Supplementary material for: Bone Bricks: The Effect of Architecture and Material Composition on the Mechanical and Biological Performance of Bone Scaffolds
Source: ACS Omega. 2022 Feb 22;7(9):7515–30. doi: 10.1021/acsomega.1c05437 (PMC8908495; doi:10.1021/acsomega.1c05437)
Supplement: Supplementary file 1 — ao1c05437_si_001.pdf [file ao1c05437_si_001.pdf]

Bone Bricks: The effect of architecture and material composition on the mechanical and biological performance of bone scaffolds

Evangelos Daskalakis[a], *Boyang Huang*[a], *Cian Vyas*[a], *Anil A. Acar*[b,c,d], *Fengyuan Liu*[f], *Ali Fallah*[b,c,d], *Glen Cooper*[a], *Andrew Weightman*[a], *Gordon Blunn*[e], *Bahattin Koç*[b,c,d], *and Paulo Bartolo*[a, *]

Number of pages: 4

Number of Tables: 2

Table S1 Morphological characteristics of bone bricks structures for different configurations.

**Material Composition**	**PCL**		**PCL/HA/TCP (80/10/10wt%)**		**PCL/TCP (90/10wt%)**		**PCL/TCP (85/15wt%)**		**PCL/TCP (80/20wt%)**		**PCL/HA (90/10wt%)**		**PCL/HA (85/15wt%)**		**PCL/HA (80/20wt%)**	
**Configuration**	Pore Size (μm)	Filament Width (μm)	Pore Size (μm)	Filament Width (μm)	Pore Size (μm)	Filament Width (μm)	Pore Size (μm)	Filament Width (μm)	Pore Size (μm)	Filament Width (μm)	Pore Size (μm)	Filament Width (μm)	Pore Size (μm)	Filament Width (μm)	Pore Size (μm)	Filament Width (μm)
Case 1	741±5	334±12	789±85	330±16	786±98	329±12	796±127	322±10	809±12	338±9	784±109	336±4	790±74	339±9	800±64	333±8
Case 2	652±110	326±12	726±104	347±9	665±85	330±15	711±113	329±4	758±95	326±6	659±129	326±7	668±135	323±5	754±123	336±4
Case 3	465±154	341±12	490±191	305±3	485±135	327±18	498±103	339±9	504±118	333±9	481±131	332±7	490±120	303±7	504±109	340±8
Case 4	647±85	344±12	684±38	327±8	683±46	338±14	698±31	318±4	708±60	335±4	682±75	333±6	694±60	344±7	704±73	328±5
Case 5	631±85	334±16	657±87	334±2	650±71	326±13	663±61	327±5	675±93	331±15	649±69	329±2	659±49	337±5	668±61	333±8
Case 6	420±111	331±9	430±52	323±3	437±60	337±10	438±77	328±9	437±58	320±9	435±77	313±5	437±42	319±4	434±75	325±5
Case 7	562±79	344±16	595±44	339±6	578±61	324±7	594±41	339±4	615±80	318±6	575±86	337±2	590±59	327±12	613±62	334±2
Case 8	448±65	356±9	509±65	340±5	454±47	338±13	474±64	332±7	568±79	329±8	450±94	330±3	471±81	333±6	564±91	326±9
Case 9	333±90	341±3	352±61	333±5	335±57	318±29	349±71	294±3	377±64	331±5	331±98	333±9	342±41	321±5	373±70	336±10

Table S2. WCA results of bone bricks structures at 0s and 20s.

**Material Composition**	PCL						PCL/TCP (90/10wt%)						PCL/TCP (85/15wt%)						PCL/TCP (80/20wt%)					
**Part**	a)		b)		c)		a)		b)		c)		a)		b)		c)		a)		b)		c)	
**Time (s)**	0	20	0	20	0	20	0	20	0	20	0	20	0	20	0	20	0	20	0	20	0	20	0	20
**Case 1**	64	49	61	48	58	47	68	65	67	65	65	64	56	54	56	54	55	53	57	55	54	52	50	50
**Case 2**	76	76	75	74	74	72	63	54	62	53	60	50	51	49	51	49	50	49	52	48	51	48	50	47
**Case 3**	76	72	74	70	71	68	62	55	62	53	60	50	49	47	48	46	47	46	57	57	56	55	53	53
**Case 4**	75	68	72	68	70	67	61	59	61	59	60	59	57	49	56	47	55	45	56	54	55	54	54	54
**Case 5**	57	55	56	54	55	53	49	48	48	48	49	47	53	48	51	45	49	45	51	47	47	45	45	45
**Case 6**	55	54	54	54	53	53	53	44	50	44	50	44	49	48	48	48	48	47	47	45	45	45	45	45
**Case 7**	59	56	58	55	56	54	55	48	55	47	55	45	53	48	53	47	52	46	58	54	57	53	55	52
**Case 8**	63	62	63	61	63	61	56	48	55	47	55	47	54	48	54	47	54	45	48	45	48	45	48	45
**Case 9**	54	53	53	53	53	53	61	60	60	58	59	57	56	51	53	50	49	45	66	66	63	63	61	60

Table S2. (cont.) WCA results of bone bricks structures at 0s and 20s.

**Material Composition**	**PCL//HA/TCP (80/10/10wt%)**						**PCL/HA (90/10wt%)**						**PCL/HA (85/15wt%)**						**PCL/HA (80/20wt%)**					
**Part**	a)		b)		c)		a)		b)		c)		a)		b)		c)		a)		b)		c)	
**Time (s)**	0	20	0	20	0	20	0	20	0	20	0	20	0	20	0	20	0	20	0	20	0	20	0	20
**Case 1**	66	66	63	61	60	58	68	41	67	40	65	38	43	29	42	29	41	29	42	30	39	27	37	23
**Case 2**	77	77	76	76	76	76	70	68	69	68	67	65	50	50	50	50	49	49	72	67	69	67	67	67
**Case 3**	78	77	78	76	78	75	65	64	65	64	64	64	52	52	52	52	52	51	98	97	96	93	94	93
**Case 4**	54	53	54	52	53	51	76	72	74	72	73	71	63	59	61	59	60	58	86	81	80	75	75	69
**Case 5**	70	70	70	69	70	68	69	67	69	66	69	66	61	58	58	55	54	51	68	68	64	64	60	59
**Case 6**	65	64	65	63	64	63	58	57	57	56	56	56	60	60	59	58	57	57	61	58	60	58	59	57
**Case 7**	74	72	72	70	69	66	57	52	57	50	56	50	66	61	63	59	60	57	68	64	66	63	65	61
**Case 8**	79	75	76	72	74	71	65	64	64	64	64	63	68	66	67	63	64	60	74	68	74	68	73	67
**Case 9**	82	79	80	78	78	76	53	52	52	50	52	50	54	54	54	53	53	53	71	71	70	70	68	68
